# Microstructural organization of superior longitudinal fasciculus and cingulum bundle support metacognition driven cognitive offloading

**DOI:** 10.1038/s41598-025-18631-5

**Published:** 2025-09-26

**Authors:** Yunxuan Zheng, Bin Bo, Danni Wang, Yiyang Liu, Lei Shi, Sam J. Gilbert, Yao Li, Sze Chai Kwok

**Affiliations:** 1 Shanghai Key Laboratory of Brain Functional Genomics, Key Laboratory of Brain Functional Genomics (Ministry of Education), School of Psychology and Cognitive Science, East China Normal University, Shanghai, 200062 China; 2 School of Psychology, Georgia Institute of Technology, Atlanta, GA USA; 3 National Engineering Research Center of Advanced Magnetic Resonance Technologies for Diagnosis and Therapy, School of Biomedical Engineering, Shanghai Jiao Tong University, Shanghai, China; 4 Phylo-Cognition Laboratory, Division of Natural and Applied Sciences, Digital Innovation Research Center, Duke Institute for Brain Sciences, Duke Kunshan University, Kunshan, 215316 China; 5 Duke Kunshan University—The First People’s Hospital of Kunshan Joint Brain Sciences Laboratory, Kunshan, 215399 China; 6 Institute of Cognitive Neuroscience, University College London, London, WC1N 3AZ UK; 7 Department of Neurosurgery, The First People’s Hospital of Kunshan, China Medical University, Kunshan, 215399 China

**Keywords:** Prospective memory, Cognitive offloading, Metacognition, DTI, Fornix, Superior longitudinal fasciculus, Cingulum bundle, Human behaviour, Cognitive neuroscience, Learning and memory, Neural circuits, Diffusion tensor imaging

## Abstract

People often use external tools to offload cognitive demands associated with remembering future intentions. While previous research has established a causal role of metacognition in cognitive offloading, the neural basis of white matter tracts supporting this metacognitive control process remains unclear. To address this, we conducted a study with 34 participants using diffusion tensor imaging (DTI) to examine how white matter connectivity supports metacognition driven cognitive offloading. Behaviorally, we replicated prior findings showing that under-confidence in internal memory predicts a bias toward using external reminders. At the neural level, we used diffusion tensor imaging to quantify fractional anisotropy (FA), a measure of microstructural integrity in white matter. We found the microstructural integrity of the superior longitudinal fasciculus (SLF) and cingulum bundle (CB) predicted deviations from the optimal use of reminders. The microstructural integrity of the fornix negatively predicted participants’ confidence in performing the task when restricted to internal memory. Our findings reveal the microstructural organization of the white-matter tracts in the fronto-temporal-parietal network are related to metacognition driven cognitive offloading. We discuss several aspects of metacognition driven cognitive offloading from a white matter microstructural perspective.

## Introduction

Prospective memory refers to the ability to remember an intention to be fulfilled under an appropriate context in the future^1–3^. Although successful fulfillment of delayed intentions constitute a substantial part of meaningful life, 50%−70% of memory failures stem from failures in prospective memory^4^. Therefore, many people choose to use external aids to support internal memorization of delayed intentions. For example, a person might write a shopping list before going to the grocery store. In cognitive science, such a strategy of using physical actions to reduce information processing requirements and cognitive demands is known as cognitive offloading^5,6^.

Given the ubiquity of cognitive offloading in everyday life, recent studies have tried to understand when and why people adapt the cognitive offloading strategy^5,7^. For instance, cognitive offloading can be considered as a preference to avoid the effort required to maintain information in prospective memory^8–11^. Consequently, memory for offloaded items tends to be relatively poor, if reminders are unexpectedly removed^12–14^. Recent evidence has also demonstrated that cognitive offloading is causally driven by metacognition, or rather specifically metamemory, the ability to monitor and control one’s own memory process^15–19^. That is, people are more likely to commit to cognitive offloading behaviors when they are under-confident about their prospective memory. In contrast, when people feel more confident in their task performance, they would be willing to exert more effort and invest more in their cognitive control^20,21^.

**Fig. 1:**
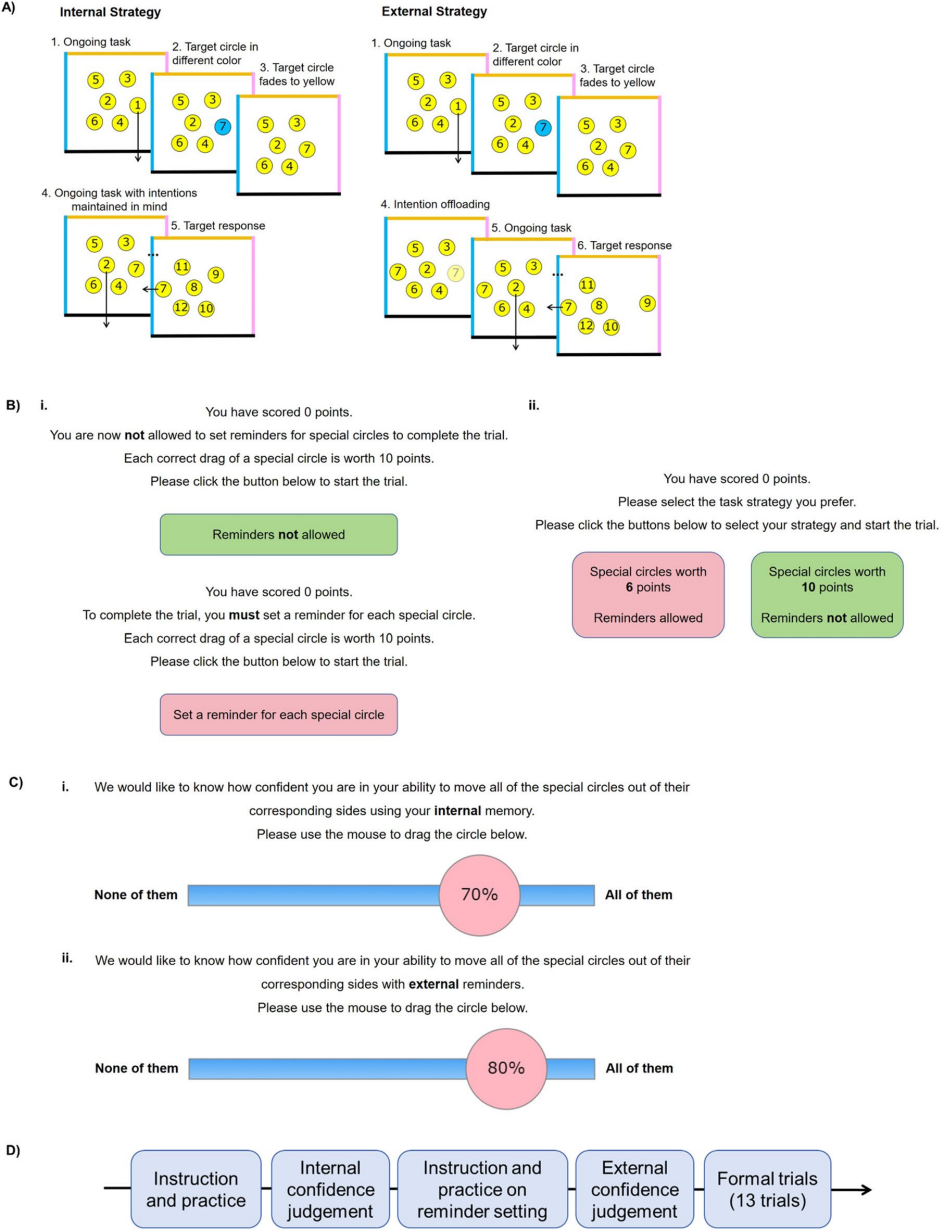
Task Procedure. **(A)** In the delayed intention task, participants perform a self-paced task where they sequentially drag numbered circles (1–17) to the borders of a box. Most circles (10/17) are yellow and need placement at the bottom border. Occasionally, a circle appears in a non-yellow colour (blue, orange, pink), corresponding to the left, top, or right borders. These target circles must be dragged to their color-matched borders when their number in the sequence is reached. Non-yellow circles turn Yellow after 2 s, requiring participants to form a delayed intention. Task points are accumulated for correct placements. In the internal strategy trials, participants use internal memory to complete the task. In the external strategy trials, participant can first drag the target circle near its corresponding border as a visual cue to remind them of the correct action. **(B)** External reminder strategy selection: (i) In 3 out of 13 trials, participants were forced to rely on internal memory, and in another 3 trials, on external reminders. Correct actions earned 10 points regardless of strategy. (ii) In the remaining 7 trials, participants freely chose between strategies. External reminder actions earned 2–8 points, while internal memory actions always earned 10 points. **C)** Post-training confidence ratings: After practice trials, participants rated their confidence in their task performance using internal memory (i) or external reminders (ii). **D)** Task event sequence: An overview of the sequence of events in the task.

We adapted a cognitive offloading task from Sachdeva and Gilbert^11^, measuring aspects of prospective memory in a way that requires remembering and acting delayed intentions (Fig. 1). In this task, participants either choose freely between using internal memory (for maximum points) or external reminders (for fewer points), or were forced to use one or the other strategy. By manipulating points in this way, we could compare each participant’s strategy choices with their optimal (point-maximising) strategy. To put this measure in terms of cognitive constructs, the reminder bias we computed here (see behavioral indices in Methods and Materials) reflects a tendency to engage in cognitive offloading compared to what would be optimal.

Neurally, previous functional neuroimaging research has highlighted the role of the rostral prefrontal cortex (rPFC) in maintaining delayed intentions, with the medial and lateral rPFC serving dissociable functions in this process^1,3,22–24^. Specifically, the medial rPFC has been hypothesized to maintain the specific details of delayed intentions, while the lateral rPFC plays a more content-free role^25^. A recent study by Boldt and Gilbert^26^ demonstrated how the cognitive offloading of setting external reminders may be driven by metamemory. They found that metamemory monitoring regions such as lateral PFC (lPFC), dorsal anterior cingulate cortex (dACC), and precuneus^27–30^ are involved in rating the confidence in remembering a delayed intention. These regions overlapped with the regions associated with generating a desire to use reminders^26^. However, little is known about how such cognitive processes are supported in the brain at the white matter microstructural level.

In the present study, we thus built upon previous functional neuroimaging findings and resorted to utilizing diffusion tensor imaging (DTI), a white matter microstructural MRI technique, to explore whether the structural integrity of white matter tracts might be related to features tested by metamemory driven cognitive offloading. DTI maps the direction and magnitude of water diffusion in brain tissue, revealing the orientation and integrity of white matter tracts. Accordingly, we are able to use the data to infer anatomical connectivity—that is, how different brain regions are physically linked via axonal bundles. Rather than working to dissociate the specific roles of each component/region known to be involved in metacognitive judgements or in cognitive offloading, our rationale is to differentiate involvements of several white matter tracts that are known to connect the key brain regions reported previously, thereby hoping to elucidate the functional anatomy by these putative brain regions and areas.

We adopted an individual difference correlation approach (i.e., correlating individuals’ behavioral index with neural index across a group of participants) on a selected set of white-matter tracts across a cohort of healthy participants. We selected a set of white matter fiber tracts, namely the superior longitudinal fasciculus (SLF), the cingulum bundle (CB), the fornix, and the uncinate fasciculus (UF). Our first candidate, the SLF, links the inferior parietal lobe to the lPFC. The lPFC has been shown to support metacognitive monitoring and control^29,31^ via SLF^32^. We thus predicted that the higher structural integrity of the SLF is associated with a lower magnitude of absolute reminder bias, or in other words an optimal use of reminders. Similarly, our second candidate, the cingulum bundle (CB), is connecting the precuneus, another well established metacognitive related region^30,33^ to the dorsal anterior cingulate cortex (dACC), a cognitive control related region^34,35^. We thus similarly hypothesized that higher structural integrity of the CB would be linked to an optimal use of reminders, or as operationally defined, a lower absolute reminder bias. Apart from tracts related to cognitive offloading related metrics, we proposed that the fornix, which is an afferent and efferent connecting fibre of the hippocampal formation to various subcortical structures that support episodic memory processes^36–41^, may be related to both the task performance when participants are forced to rely on internal memory^42–44^ and to the confidence prediction on their own task performance^45^. Finally, our fourth candidate, the uncinate fasciculus (UF), connects the memory-related temporal lobe including hippocampus to the rPFC, a metacognition related region that is reported to support error monitoring in memory retrieval^46,47^. It is possible that greater structural integrity of the UF may be associated with sensitivity to one’s own errors, leading to lower confidence and lower metacognitive bias (i.e., more underconfident in the task performance with internal memory).

## Results

### Behavioral results

**Fig. 2:**
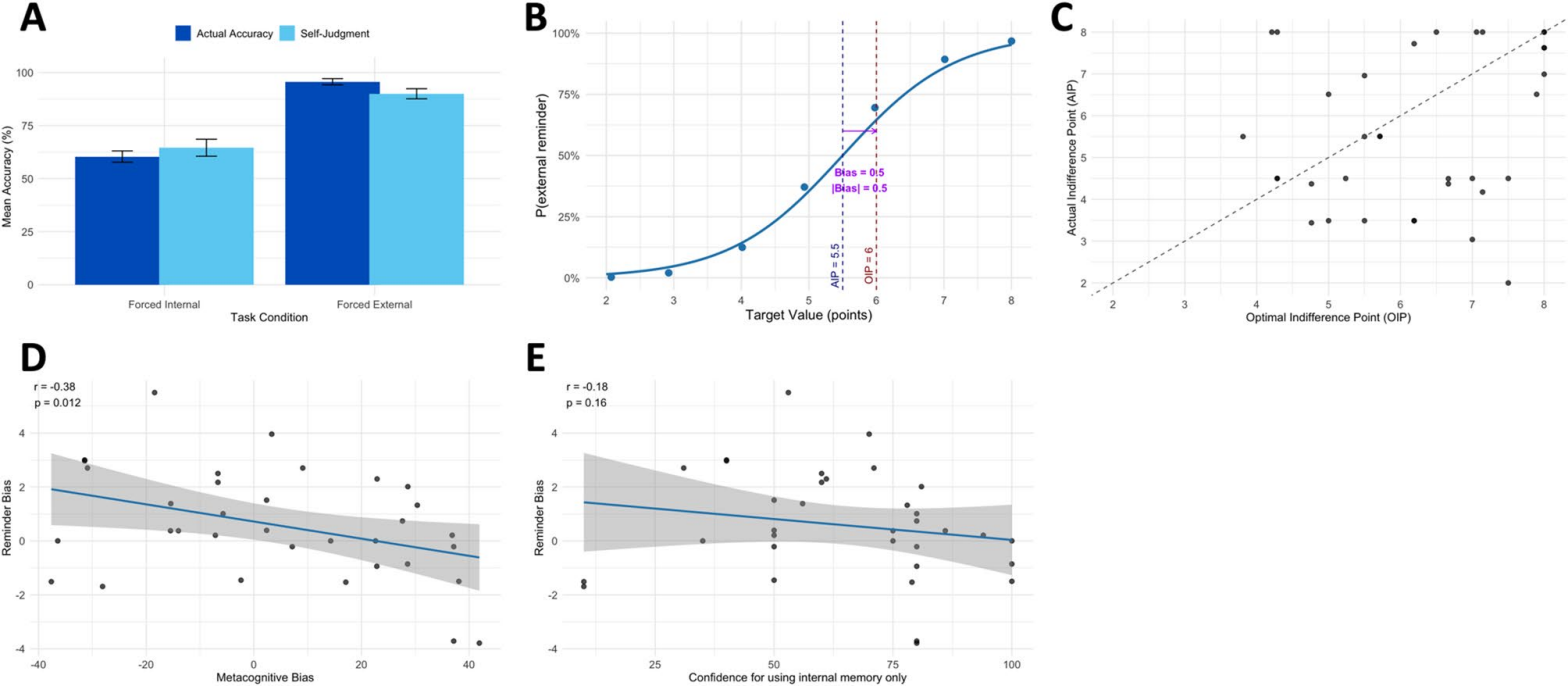
Metacognitive judgments of accuracy, computation of reminder bias using optimal indifference point (OIP) and actual indifference point (AIP), and relationship between reminder bias and metacognitive bias or self-judged accuracy (the confidence judgment) across participants. (A) Mean confidence judgments (self-reported accuracy estimates) and actual accuracy under forced-internal memory versus forced‐external reminder conditions. (B) Illustration of reminder bias computation: the probability of choosing reminders across target values (2–8) is plotted with the optimal indifference point (OIP) and the actual indifference point (AIP). Reminder bias (shorten as |bias| in the plot) is quantified as the difference between OIP and AIP. (C) Scatterplot of all participants’ AIP versus OIP. The diagonal line denotes perfect calibration (AIP = OIP); points below it indicate over‐reliance on reminders (AIP < OIP), points above indicate under‐use of reminders (AIP > OIP). The group-level deviation from this line reveals a significant reminder bias. (D) Correlation plot of the relationship between reminder bias and metacognitive bias, showing that participants who underestimate their memory accuracy (negative values on metacognitive bias) tend to rely more on reminders (positive values on reminder bias). (E) Correlation plot showing the relationship between reminder bias and the self-judged accuracy (the confidence judgment) in executing delayed intentions with internal memory.

We first investigated if there was a significant difference in accuracy between the forced internal memory and the forced external reminder conditions. We ran a 2 × 2 ANOVA and found that participants’ actual accuracy and confidence judgments were both higher when participants were forced to use external reminders than when they relied solely on internal memory (F(1, 128) = 41.21, *p* <.001; Fig. 2A). Post-hoc tests confirmed that external reminders indeed increased the delayed intention task performance (*M* = 95.6583, *SD* = 8.4020) compared to the forced internal memory condition (*M* = 60.3642, *SD* = 15.54; *t*(50.775) = 11.649, *p* <.001). There was no main effect of measures (actual accuracy/self-judged accuracy, F(1, 128) = 0.059, *p* =.808) or interaction effect between types of conditions and measures (F(1, 128) = 0.61, *p* =.436), suggesting that participants’ confidence prediction matched their actual performance in both conditions.

To quantify participants’ strategic preferences, we compared the *optimal indifference point* (OIP) and the *actual indifference point* (AIP). The OIP reflects the value at which an unbiased, reward-maximizing agent should be indifferent between using internal memory and external reminders. In contrast, the AIP denotes the actual value at which participants behaviorally exhibited indifference between the two strategies. Previous studies have consistently shown that people tend to set external reminders to assist their task performance more often than is optimal, such that the OIP is greater than the AIP^11,16,17,48,49^. On the basis of this strong and well-replicated directional prediction, we employed a one-sided paired t-test to assess reminder bias (OIP – AIP). Consistent with prior findings, we found that the discrepancy between OIP and AIP was significantly greater than zero (*M* = 0.5860, *SD* = 2.0357; *t*(33) = 1.6785, *p* =.0257). This statistical effect is reflected by the more dense individual dots below the diagonal line in Fig. 2C.

Finally, existing research has also consistently shown that the deviations in the optimal use of external reminders over internal memory (i.e., the reminder bias) was driven by the miscalibration between self-judged and actual task performance in using internal memory such that people would have stronger reminder bias when their confidence ratings are lower than their actual accuracy^11,16,17,48,49^. Therefore, guided by this empirical finding, we conducted a one-sided Pearson correlation test, and confirmed a significant negative correlation between reminder bias and the metacognitive bias for internal memory (*r*(32) = − 0.384, *p* =.0125; Fig. 2D). A cautionary note is that metacognitive bias is derived from the difference between self-judged accuracy and actual memory accuracy. Although this behavioural index already accounts for both self-judged and actual accuracy, one could still speculate that self-judged accuracy (memory confidence) alone is a plausible factor correlative with reminder bias. To test this hypothesis, we ran another one-sided correlation test for the relationship between self-judged accuracy and reminder bias. We found that reminder bias is not significantly related to participants’ overall confidence in successfully executing delayed intentions using internal memory (r(32) = –0.178, *p* =.157; Fig. 2E). That is, although there is a numerical trend, self-judged accuracy (confidence) itself is not enough driving the reminder bias. Taken altogether, we conclude that the metacognitive bias, that is, the miscalibration between self-assessed and actual memory performance, is the driver of suboptimal offloading behavior.

### DTI tractography and relationship with behavioral metrics

We then moved on to examine the relationship between these behavioral metrics and the participants’ white matter tract properties. Because the reminder bias was correlated with the discrepancy between self-estimated and actual performance under forced‐internal memory (the metacognitive bias), here we focused our analyses on metacognitive measures from the forced-internal memory condition. Given that the cognitive processes are often modulated by other factors, in all the following regression models, we input participants’ age, gender, and total intracranial volume as regressors of no interest to control for any potential influence on the regression results^50^. Within each regression analysis, the outcome and all continuous predictors (i.e., except gender) were scaled in order to better compare the coefficients across models (see *Methods and Materials* for model details).

We examined the relationship between the structure integrity of white matter (WM) tracts (indexed by fractional anisotropy; FA) and participants’ behavioral indices relevant to the metacognition driven cognitive offloading processes. FA reflects the microstructural integrity of white matter. High FA values suggest organized, healthy fiber tracts. The microstructural integrity within the superior longitudinal fasciculus (SLF), cingulum bundle (CB), and the fornix are implicated in relation to several aspects of participants’ performance in the cognitive offloading task. We did not obtain any significant results regarding the uncinate fasciculus (UF).

**Fig. 3:**
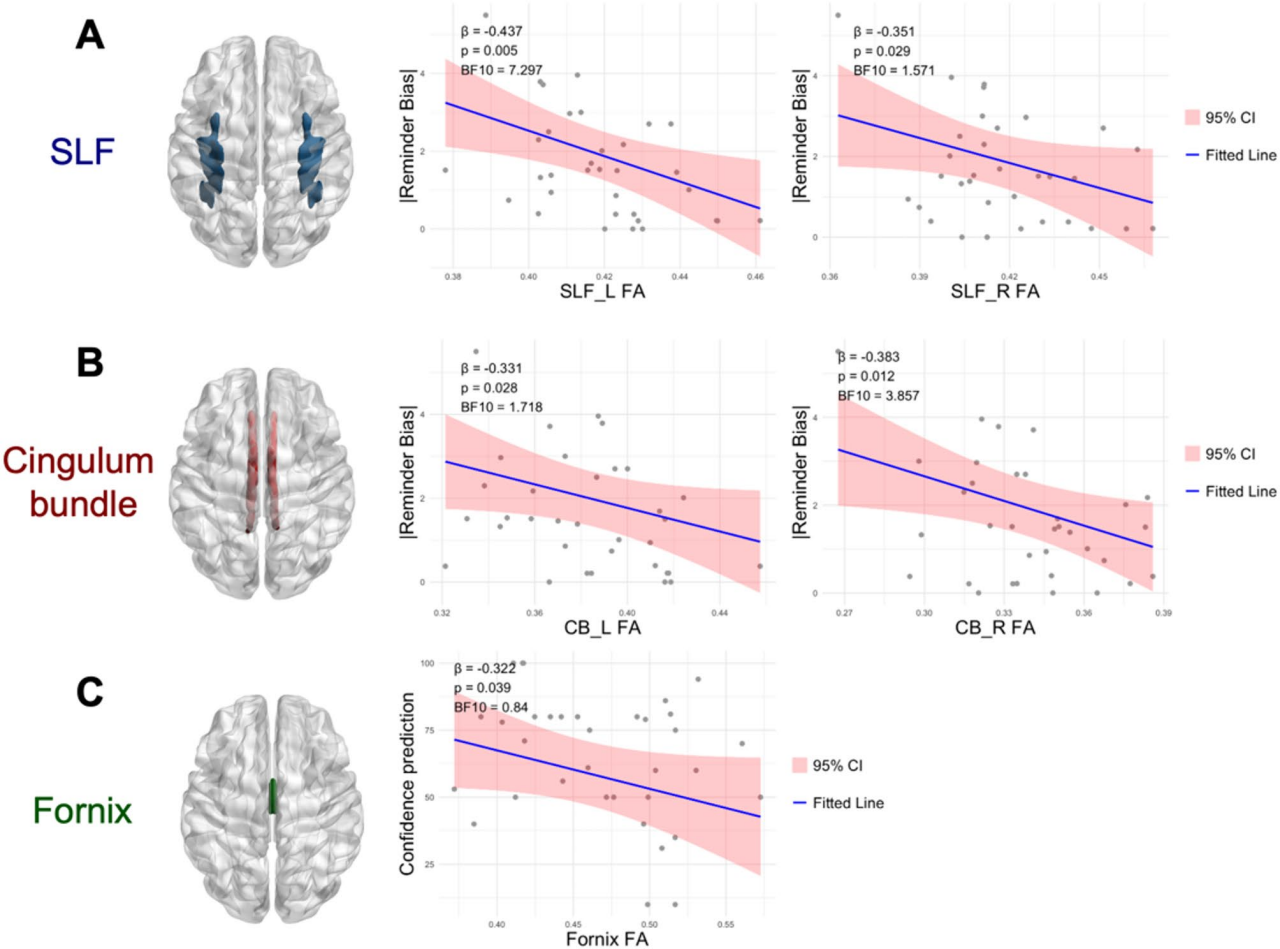
Correlations between behavior and DTI indices. (A) Fractional anisotropy (FA) of the bilateral superior longitudinal fasciculus (SLF) was negatively associated with the absolute degree of deviation from the optimal strategy choice (|reminder bias|). (B) Similarly, FA of the bilateral cingulum bundle (CB) negatively predicted the absolute bias in strategy selection (|reminder bias|). (C) FA of the fornix was negatively associated with confidence levels when participants were required to rely solely on internal memory to perform delayed intention tasks. Regression coefficients (ß), P-values, and Bayes Factors are reported in top left within each regression plot. CI = 95% confidence interval. L = left hemisphere, R = right hemisphere.

Firstly, the FA in the left and right SLF negatively predicted the absolute degree of reminder bias (or deviation) in utilizing external reminders over internal memory (SLF L: ß = −0.437, *SE* = 0.159, t(29) = −2.753, *p*_*uncorrected*_ = 0.005, *p*_*FWE*_ = 0.030, BF_10_ = 7.786; SLF R: ß = −0.351, *SE* = 0.178, t(29) = −1.972, *p*_*uncorrected*_ = 0.029, *p*_*FWE*_ = 0.175, BF_10_ = 2.302, Table 1; Fig. 3A). These findings indicate that greater structural organization in the bilateral SLF was associated with a more optimal selection between external reminders and internal memory strategies.

Regarding the cingulum bundle, the microstructural integrity of the CB negatively also predicted the absolute degree of reminder bias towards using external reminders over internal memory (CB L: ß = −0.331, *SE* = 0.166, t(29) = −1.999, *p*_*uncorrected*_ = 0.028,, *p*_*FWE*_ = 0.165, BF_10_ = 2.038; CB R: ß = −0.383, *SE* = 0.160, t(29) = −2.391, *p*_*uncorrected*_ = 0.012,, *p*_*FWE*_ = 0.071, BF_10_ = 4.291, Table 1; Fig. 3B). In other words, higher structural organization in the right CB corresponded to a more optimal strategy choice. Incidentally, the FA in the right CB also negatively predicted the degree of bias in setting up reminders (ß = −0.375, *SE* = 0.171, t(29) = −2.198, *p*_*uncorrected*_ = 0.018, *p*_*FWE*_ = 0.108, BF_10_ = 3.043), which implies a reduced reliance on external reminders for those participants with more intact CB organization.

Thirdly, the microstructural integrity of the fornix negatively predicted the level of confidence in performing the offloading task with internal memory (ß = −0.322, *SE* = 0.176, t(29) = −1.827, *p*_*uncorrected*_ = 0.039, *p*_*FWE*_ = 0.117, BF_10_ = 1.427; Table 1; Fig. 3C). This negative relationship implies, following the practice trials, participants with greater fornix microstructural organization adopt more conservative confidence predictions of their performance with respect to their internal memory. Furthermore, the fornix FA was found not correlated with the actual performance with internal memory only (ß = −0.08, *SE* = 0.188, t(29) = −0.441, *p*_*uncorrected*_ = 0.663, *p*_*FWE*_ = 0.994, BF_10_ = 0.441) nor with the metacognitive bias in calibrating between confidence and actual performance (ß = −0.25, *SE* = 0.180, t(29) = −1.406, *p*_*uncorrected*_ = 0.085, *p*_*FWE*_ = 0.255, BF_10_ = 0.843).

We did not find any of the tracts to be associated with either metacognitive bias or the absolute magnitude of metacognitive bias in this task context. The regression results are summarized in Table 1.

**Table 1:** |reminder|MetaRegression coefficients between behavioral metrics and white matter tract FA index. Statistical significant coefficients are in bold, with ** indicating p-value < 0.01, * indicating p-value < 0.05, without family-wise error correction. Linear regression analyses were conducted where age, gender, and total intracranial volume on DTI metrics were controlled. Meta bias = metacognitive bias. |Meta Bias| = absolute value of metacognitive bias; |Reminder Bias| = absolute value of reminder bias.

	Confidence Prediction (using internal memory)	Meta Bias	Reminder Bias	|Meta Bias|	|Reminder Bias|
*Superior longitudinal fasciculus*					
Left	0.152	0.059	−0.194	−0.019	−0.437**
Right	−0.016	−0.025	−0.269	0.027	−0.351*
*Cingulum bundle*					
Left	0.128	0.128	−0.264	0.030	−0.331*
Right	0.011	0.052	−0.375*	−0.085	−0.382*
*Fornix*					
	−0.322*	−0.254	−0.069	−0.148	−0.050
*Uncinate fasciculus*					
Left	0.058	0.121	0.030	−0.039	−0.158
Right	0.085	0.122	0.105	0.211	0.021

## Discussion

We often observe that in everyday life people use external reminders to offload memory demands. This can increase the amount of information stored and assist retrieval at the appropriate moment^16^. From previous fMRI studies, a number of higher cognitive regions have been involved in metacognitive monitoring and control, including the lPFC^29,31,32^, the precuneus^30,33^, and the dorsal anterior cingulate cortex (dACC)^34,35^. Since these regions’ activation levels are linked to metacognitive processes, a widespread parietal-frontal neural network^26^ is functionally engaged for metacognitive monitoring and control processes. However, their functional anatomy, especially at the white matter microstructural level, remains understudied. We therefore utilized an anatomical neuroimaging technique, diffusion tensor imaging (DTI), to examine the relationship between white matter tract microstructural integrity and metrics embedded in metacognition driven cognitive offloading.

We found evidence highlighting how white matter structural organization in the superior longitudinal fasciculus (SLF) and cingulum bundle (CB) could predict deviations from optimal use of reminders. Anatomically, the SLF connects the lateral prefrontal cortex (lPFC) and the inferior parietal lobe (IPL). Prior studies have indicated that enhanced integrity of certain portions of the SLF is linked to better metacognitive monitoring across perceptual and mnemonic domains^32^. Similarly, stronger CB integrity negatively predicted the magnitude of bias toward reliance on reminders. People with weaker CB organizations tend to rely more excessively on external reminders. The CB links the dorsal anterior cingulate cortex (dACC) with parietal regions, including the precuneus, both of which are associated with metacognitive monitoring^26,28,30,51^. These findings imply that more efficient transmission of information due to the better microstructural organization through the SLF and CB seem to facilitate the use of metacognitive signals to guide offloading behavior. This aspect of cognitive offloading is considered as metacognitive control. Therefore, the current findings on these tracts, especially those on the SLF, extend on extant functional neuroimaging evidence that at the white matter microstructural level, monitoring and control processes might also share common anatomical and neural substrates^26^.

Compared to conventional metacognition research using trial-by-trial retrospective confidence ratings, here we asked participants to indicate their confidence prospectively on their task performance after the practice and training stages. This prospective confidence judgment can be interpreted as a global confidence judgment, which reflects broader, trait-like assessments of cognitive ability. Some studies suggest that global confidence judgments are derived from and constantly updated by trial-by-trial retrospective decision confidence^52–54^, which correspond to our practice and training stages. We found that structural organization of the fornix negatively predicted confidence predictions about one’s own subsequent memory task performance. Importantly, this relationship was not observed for actual memory performance. This negative result thus helps dissociate the fornix’s involvement in memory vs. error monitoring/performance assessment (confidence prediction) in this context. A possible account is that participants with greater fornix integrity possess heightened sensitivity to errors committed during practice trials, via better hippocampal outputs to the ventral striatum^36^. With richer error related information in the ventral striatum, a region known to encode prediction-error signals and to track global confidence on task performance^51^, these participants may question their forthcoming task performance and thereby lower their confidence prediction. This interpretation is speculative, but our results may provide insights into the white matter microstructure underlying the global confidence judgments. Given that the fornix is an afferent and efferent of the hippocampal formation, the finding agrees with other recent work reporting that the hippocampus is implicated in mnemonic metacognition^45^. In contrast with the fornix, SLF and CB primarily support transmission of the metacognitive signal and is related to dynamic, moment-to-moment metacognitive monitoring. This could explain why SLF and CB structural organization did not predict the level of confidence ratings nor metacognitive bias in our study.

One may consider how our experimental paradigm and findings could be generalized to real-world prospective memory scenarios. There are indeed differences. For example, it is rare for anyone to have to remember a list of 17 shopping items (see Fig. 1) and for anyone to do it 13 times (trials) consecutively all in a go. A more comparable scenario would be like a husband (or wife) was instructed by their partner to go get a long list of items before they left home. The requester would name the items (often verbally) one by one, which is more akin to the paradigm here wherein the item was presented one by one. It is up to the recipient of the request (the tasked person) to decide whether they would write the items down on a piece of paper during the process of receiving the verbal request. The likelihood of such an act would thus be what we operationally defined as reminder bias. We thus argue that our paradigm carries ecological relevancy. A nuanced difference remains though, that our experimental paradigm was self-paced whereas in the real-life husband-and-wife scenario, the requestor would go through the list at their whim, often non-stop between items. The key aspect here is on the tasked person’s tendency to resort to offloading and their related metacognitive bias, which can be measured in both scenarios above.

Although the Bayesian power analysis indicates that a sample size of 34 participants provides sufficient power to detect a small to medium effect size in a single multiple linear regression, it does not address the issue of multiple comparisons in the study. We acknowledge that the statistical power becomes more limited when conducting several regression analyses. Indeed, apart from the relationship between the absolute degree of reminder bias and left SLF FA, none of the other reported significant effects survived correction. However, Bayes factor analyses provided complementary evidence: we observed substantial support (BF₁₀ > 3) for relationships between absolute reminder bias and both left SLF FA and right cingulum bundle FA, as well as between reminder bias and right cingulum bundle FA. Taken together, results reported in this study should be interpreted with caution given the increased potential for false positives in the context of multiple comparisons. Nevertheless, as the first study to explore how white matter connectivity supports metacognition-driven cognitive offloading, we believe our findings—though based on a modest sample size—remain valuable in generating specific hypotheses for future research. With a larger sample size, future research could also investigate whether and how individual differences—such as age, gender, and psychiatric symptoms—influence metacognition driven cognitive offloading strategies through differences in brain microstructural properties^48,55–58^. Incidentally, recent studies have shown that individuals possess a meta-metacognitive ability, namely, the capacity to evaluate the quality of their own confidence judgments^44,59^. Future work could therefore examine which white matter tracts also support meta-metacognition and how this capacity influences the optimal use of external reminders in prospective memory tasks.

In summary, our findings identify fronto-temporal-parietal white-matter tracts related to several metrics measured by metacognition driven cognitive offloading in a prospective memory task. These cognition-microstructural findings provide a link reflecting the relationship between cognitive offloading and the organization of white matter microstructures.

## Methods and materials

### Participants

38 adult participants were recruited through local advertisement. All participants had normal or corrected-to-normal vision, reported no history of no color blindness, psychiatric and neurological diseases, and no other contraindications for MRI. All participants provided written informed consent. The study was ethically approved by the Shanghai Jiao Tong University Institutional Review Board. All procedures were performed in accordance with the institutional guidelines.

Three participants were removed because they did not attend the MRI scanning. In line with the exclusion criteria used by Gilbert et al.^17^, we further excluded one participant who had a negative correlation between target value and likelihood of choosing to use reminders, implying a random or counter-rational strategy choice behavior. Therefore, 34 participants (19 females, mean age $\:\pm\:$ SD = 28.47 $\:\pm\:$ 8.82) were taken into analysis. Our Bayesian power analysis indicated that a sample size of 34 subjects provided high power to detect a medium effect size (regression coefficient of 0.5) in each of our planned subject-level multiple regression analyses.

### Task procedure

In the following, we described three key stages of the task: Delayed intention, external reminder strategy selection, and pre-task confidence ratings.

#### Delayed intention

Participants engaged in a cognitive offloading task adapted from Gilbert et al.^17^. In this task, circles numbered from 1 to 17 would be presented within a box with colored borders. The primary objective for participants was to sequentially drag each circle to the bottom border in ascending numerical order (Fig. 1A). At the beginning of each trial, six Yellow circles with numbers 1 to 6 were randomly positioned inside the box. Participants were instructed to drag these Yellow circles to the bottom of the box. When a circle reached the box bottom, a new circle would replace it in its original location. For instance, dragging the circle numbered 1 to the bottom would be followed by the appearance of a circle numbered 7, taking its place.

Among the 17 circles per trial, most (10 out of 17) appeared in Yellow and were to be dragged to the bottom edge as usual. However, on each trial, 7 of these circles initially appeared in a non-yellow color—blue, orange, or pink—corresponding to the left, top, or right edge of the box. These were designated as target circles. After 2 s, each colored target circle faded to Yellow to match the others. Participants were instructed to remember both the digit and the original color of these target circles and to drag them to their color-matched edge when their digit was reached in the ongoing sequence. For example, after dragging Circle 1 to the bottom, an orange Circle 7 might appear and fade to Yellow after 2 s; the participant must then continue dragging Circles 2–6 in order, while remembering to drag Circle 7 to the orange edge once it is reached.

This design required participants to maintain multiple delayed intentions — actually up to 6 at a time — throughout a sequence. This exceeded typical short-term memory capacity, as indicated by accuracy well below ceiling (approximately 60%) when participants relied on their own memory, hence there was a clear improvement in performance when reminders were used. Moreover, we also randomized both the digits involved and target directions across trials, precluding fixed verbal encoding strategies (e.g., rote rehearsal like “digit seven-top, digit nine-left”). For illustration, we set up a link for a demo version of the task to help readers to try the task out for themselves: https://cognitiveoffloading.net/ZhengDemo. This is exactly the task from the real experiment we ran, except that it does not store any data and we have replaced all the Chinese instructions with the original English version from Sam Gilbert’s laboratory. For the overall structure of the paradigm, see Fig. 1D.

#### External reminder strategy selection

In addition to maintaining the delayed intention, participants were instructed how to set an external reminder for task performance. In such instances, participants needed to promptly drag a target circle near its corresponding box border upon appearance. For example, if a blue circle appeared, participants should immediately drag it close to the left border. This ensured that when the circle number was reached in the sequence, its location served as a reminder for participants to fulfill the intended action.

Each participant underwent 13 trials. In three trials, participants were explicitly instructed to rely solely on their internal representation of the intention, while in another set of three trials, participants were informed to rely exclusively on external reminders. In these forced-choice trials, every correct drag of a target circle resulted in a 10-point increase, regardless of the chosen strategy (Fig. 1B i). However, in the remaining seven trials, participants were given the freedom to choose their strategy (internal memory or external reminders) (Fig. 1B ii). Importantly, the points awarded for each correct drag varied based on the chosen strategy. Opting for internal memory yielded 10 points per accurate drag, while choosing external reminders resulted in a variable point range (2–8, randomly selected for each trial) for each correct drag, which was lower than the internal memory strategy. Therefore, simply always choosing to use reminders would not be an optimal strategy for task performance. Instead, participants have to balance the higher number of points when remembering with internal memory against the greater chance of success when using external reminders.

#### Post-training confidence ratings

Before the proper trials, participants underwent practice trials to familiarize themselves with the task and the two strategies. Following these practice trials, participants provided two separate confidence ratings (i.e., the prospective confidence) in task accuracy using internal memory (Fig. 1C i) or external reminders (Fig. 1C ii) respectively.

### Behavioral indices

We used the following behavioral indices to quantify several aspects of each participant’s task performance. The first three are of particular interest in our present investigation in relation to the microstructural integrity of white matter tracts, whereas the next four were for replication of previous behavioral findings^11,17,48,55^ and to derive some of our key behavioral metrics.

*Reminder bias* reflects one’s deviation from optimal reminder use. A value at zero indicates that a participant is optimal in selecting between internal memory and external reminder strategies. We also computed the absolute (unsigned) value of reminder bias (|Reminder Bias|) to capture the absolute magnitude of deviation from optimal reminder use. For example, consider a participant who scores 50% accuracy when using their own memory and 100% accuracy when using reminders. If this participant was offered the choice between scoring 10 points for correct responses with internal memory (10 points x 50% = 5 points) or 6 points when using external reminders (6 points x 100% = 6 points), the optimal (point-maximising) strategy is to choose reminders. However, if they are only offered 4 points when using external reminders then the optimal strategy is to use internal memory (4 points x 100% = 4 points). By comparing each participant’s strategy choices with their individually-computed optimal strategy, we calculated whether they exhibited a pro- or anti-reminder bias. Mathematically, it is computed as the difference between Optimal indifference point (OIP) and Actual indifference point (AIP). An unbiased participant’s AIP should align with the OIP. Otherwise, a higher OIP than AIP indicates a bias toward external reminders because the participant would set up reminders even when the reward associated with the target is less than optimal. Conversely, a higher AIP than OPI would indicate a bias toward using internal memory (see below and Fig. 2B-C).

*Confidence judgment*, participants’ own estimation on their likelihood that they can successfully move all the target circles by using internal memory only.

*Metacognitive bias*, the subtraction between confidence judgment and the objective percentage of targets remembered when using internal memory (i.e., the internal accuracy). In terms of cognitive constructs, metacognitive bias reflects the extent to judge one’s own performance to be better or worse than it really is. The degree of metacognitive bias reflects how much a participant was over- or under-confident about their ability to remember targets with their internal memory. Negative values indicate under-confidence and vice versa, and a value at zero indicates that the participant is perfectly calibrated between their confidence prediction and actual task performance. We computed the absolute (unsigned) value of metacognitive bias (|Meta Bias|) to reflect the absolute magnitude of miscalibration in confidence judgments.

The following four were not of our main focus but were used to derive the three aforementioned metrics and for replication of previous behavioral findings.

*Internal accuracy* (ACC_FI_), the average percentage of target circles correctly moved to designated locations in trials where participants were required to rely solely on internal memory (i.e., forced internal trials).

*External accuracy* (ACC_FE_), the average percentage of target circles correctly moved to designated locations in trials where participants were required to rely solely on external reminders (i.e., forced external trials).

*Optimal indifference point* (OIP), the value of target circles at which an unbiased, reward-maximizing participant should be indifferent towards either the internal strategy or external strategy. OIP is calculated as 10*ACC_FI_/ACC_FE_. Suppose a participant can accurately remember 60% of targets with internal memory (ACC_FI_ = 0.6) and all targets with external reminders (ACC_FI_ = 1), then in this case the OIP value should be 6. That is, obtaining 6 points per target with external reminders would lead to the same amount of points as obtaining 10 points per target with internal memory. When the value of a target circle is above 6, it would be optimal to choose external reminders, as it maximizes the rewards. Similarly, the value of a target circle is below 6, it would be optimal to choose internal memory.

*Actual indifference point* (AIP), the actual value of target circles at which participants showed indifference. The AIP is determined by assessing the probability of choosing an external strategy over an internal one across all external target values, and was obtained by fitting a psychometric function using the R package “*quickspy.*”

### DTI image acquisition

All participants underwent magnetic resonance imaging (MRI) on a 3-tesla system equipped with a standard 32-channel head and neck coil (uMR790, United Imaging, Shanghai, China). The MRI protocol included the following sequences: (1) 3D T_1_-weighted gradient-recalled echo sequence with repetition time (TR) = 8.1 ms, echo time (TE) = 3.4 ms, inversion time (TI) = 1060 ms, matrix size = 320 × 300 × 208, voxel size = 0.8 mm isotropic, flip angle = 8°, bandwidth = 260 Hz/pixel, acceleration factor = 2, and field of view (FOV) = 256 × 240 mm². (2) Multi-shell diffusion-weighted imaging (DWI) using multi-band accelerated echo planar imaging (EPI): 96 diffusion-weighted directions, including 32 directions at b = 1000 s/mm² and 64 directions at b = 2000 s/mm², along with 4 b = 0 images. The acquisition parameters for the DWI included: voxel size = 1.5 mm, TR = 5150 ms, TE = 77 ms, FOV = 210 × 210 mm², and flip angle = 90°.

### DTI data processing

The multi-shell diffusion magnetic resonance imaging (dMRI) data underwent preprocessing for denoising and removal of Gibbs artifacts utilizing tools from MRtrix 3.0 (http://www.mrtrix.org). Following this, head-motion correction and eddy current correction were carried out using the Functional Magnetic Resonance Imaging of the Brain Software Library (FSL; v6.0; https://fsl.fmrib.ox.ac.uk/fsl/fslwiki). Subsequently, the diffusion tensor fitting for all diffusion shells with b-values up to 1000 s/mm² was performed using the *dtifit* function within FSL. The fractional anisotropy (FA) images were obtained for each participant.

White matter tracts of interest—including the column and body of the fornix, bilateral uncinate fasciculus (UF), cingulum bundle, and superior longitudinal fasciculus (SLF)—were delineated based on the ICBM-DTI-81 white matter labels atlas. To accurately register these masks onto the FA maps, diffusion-weighted images were first co-registered to T1-weighted structural images and subsequently normalized to Montreal Neurological Institute (MNI) space using the transformation matrix obtained from structural image normalization. The inverse of these transformations was then applied to the masks to align them within the native diffusion-weighted image space. All registration procedures were executed using Advanced Normalization Tools (ANTs, http://stnva.github.io/ANTs/). T1-weighted structural images for each participant first underwent intensity non-uniformity correction using N4 bias field correction (ANTs). Subsequently, tissue segmentation was performed using FreeSurfer (version 7.4.1; https://surfer.nmr.mgh.harvard.edu/). The resulting individual white matter (WM) masks were co-registered to the diffusion space and used to refine the atlas-based ROIs by intersection, in order to reduce potential registration inaccuracies and minimize partial volume effects from cerebrospinal fluid (CSF). The mean values of FA were then computed for each subject’s fornix, UF, cingulum bundle, and SLF, incorporating all voxels within the respective tracts. The FA is interpreted as a quantitative biomarker of white matter ‘integrity’^60,61^. We will use this biomarker for our investigation of cognitive processes.

### Statistical analysis and code availability

The behavioural measure of reminder bias was calculated by subtracting the Actual Indifference Point (calculated with the R “quickpsy” package) from the Optimal Indifference Point. All the ANOVA, pairwise t-tests and linear regression analyses in this study were conducted using the R “stats” package. In the regression analyses, in addition to examining the relationship between a given white matter tract FA value and a given behavioral index, we also control for the influence of participants’ age, gender, and total intracranial volume (TIV). All the continuous outcome and predictors were firstly scaled, in order to better compare the coefficients across models. The model did not contain any factors of random effects because we input single-observation-per-subject data in that aspect. Specifically, the regression model was defined as follows:

FA of a white matter tract of interest (scaled) ~ A behavioral index of interest (scaled) + Age (scaled) + Gender + TIV (scaled).

Because the purpose of the analyses is to investigate whether any behavioural index would relate to each white matter tract microstructure, to control family-wise error within each tract, we applied a Bonferroni correction across those behavioral indexes of interest. We chose Bonferroni over alternative approaches (e.g., FDR) because the number of comparisons per tract is modest (six), making the conservativeness of Bonferroni less problematic while ensuring that the reported p-values after correction remain easily interpretable and reproducible. We used the R “BayesFactor” package to compute inclusion Bayes factors for each analysis, by comparing the full model including the behavioral predictor with a reduced model containing only the control variables.

To evaluate the sensitivity of our study design, we also performed a Bayesian power analysis with the R “BayesFactor” package to determine whether the present study has sufficient power to detect a small to medium effect size. In each of 1,000 Monte Carlo iterations, we generated a synthetic dataset of 34 subjects. To mimic the distributions of variables after z-scoring in the planned analyses, the primary predictor and the continuous control variables (age and total intracranial volume, TIV) were sampled independently from a standard normal distribution (mean = 0, SD = 1), while gender was simulated as a binary variable with equal probability. The outcome variable was then constructed as a linear combination of these predictors: the predictor of interest was assigned a true standardized effect size of β = 0.35, the age and TIV covariates were assigned smaller coefficients (β = 0.10), and gender contributed a fixed binary offset. To reflect unexplained variability, Gaussian noise was added to the outcome with SD = 0.50 on the standardized scale, representing random error and measurement imprecision. For each dataset, we fit two Bayesian linear models using the “BayesFactor” R package: a full model including the predictor and controls, and a reduced model including only the controls. The inclusion Bayes factor was computed by comparing these models, and power was defined as the proportion of simulations in which the Bayes factor exceeded 3 (moderate evidence for the predictor). Under these assumptions, the estimated Bayesian power was 0.903, indicating that with 34 subjects the design is well-powered to detect a medium standardized effect of 0.35. We set the effect size at 0.35 as a realistic benchmark because associations between behavioral measures and structural neuroimaging indices often fall in the small-to-moderate range (*r* ≈.3–0.4)^62–64^.

The preprocessed behavioral and DTI data, along with all relevant analysis code, are available at https://osf.io/jdnk4/.

## Data Availability

Data and materials are available at https://osf.io/jdnk4/.
